# Involvement of TGF-β and Autophagy Pathways in Pathogenesis of Diabetes: A Comprehensive Review on Biological and Pharmacological Insights

**DOI:** 10.3389/fphar.2020.498758

**Published:** 2020-09-15

**Authors:** Fatemeh Heydarpour, Soraya Sajadimajd, Elahe Mirzarazi, Pouya Haratipour, Tanuj Joshi, Mohammad Hosein Farzaei, Haroon Khan, Javier Echeverría

**Affiliations:** ^1^Medical Biology Research Center, Health Technology Institute, Kermanshah University of Medical Sciences, Kermanshah, Iran; ^2^Departament of Biology, Faculty of Sciences, Razi University, Kermanshah, Iran; ^3^Institute of Biochemistry and Biophysics, University of Tehran, Tehran, Iran; ^4^Department of Chemistry, Sharif University of Technology, Tehran, Iran; ^5^PhytoPharmacology Interest Group (PPIG), Universal Scientific Education and Research Network (USERN), Los Angeles, CA, United States; ^6^Department of Pharmaceutical Sciences, Faculty of Technology, Kumaun University, Nainital, India; ^7^Pharmaceutical Sciences Research Center, Kermanshah University of Medical Sciences, Kermanshah, Iran; ^8^Department of Pharmacy, Abdul Wali Khan University, Mardan, Pakistan; ^9^Departamento de Ciencias del Ambiente, Facultad de Química y Biología, Universidad de Santiago de Chile, Santiago, Chile

**Keywords:** diabetes, autophagy, TGF-β, natural agents, systematic review

## Abstract

Despite recent advancements in clinical drugs, diabetes treatment still needs further progress. As such, ongoing research has attempted to determine the precise molecular mechanisms of the disorder. Specifically, evidence supports that several signaling pathways play pivotal roles in the development of diabetes. However, the exact molecular mechanisms of diabetes still need to be explored. This study examines exciting new hallmarks for the strict involvement of autophagy and TGF-β signaling pathways in the pathogenesis of diabetes and the design of novel therapeutic strategies. Dysregulated autophagy in pancreatic β cells due to hyperglycemia, oxidative stress, and inflammation is associated with diabetes and accompanied by dysregulated autophagy in insulin target tissues and the progression of diabetic complications. Consequently, several therapeutic agents such as adiponectin, ezetimibe, GABA tea, geniposide, liraglutide, guava extract, and vitamin D were shown to inhibit diabetes and its complications through modulation of the autophagy pathway. Another pathway, TGF-β signaling pathway, appears to play a part in the progression of diabetes, insulin resistance, and autoimmunity in both type 1 and 2 diabetes and complications in diabetes. Subsequently, drugs that target TGF-β signaling, especially naturally derived ones such as resveratrol, puerarin, curcumin, hesperidin, and silymarin, as well as Propolis, *Lycopus lucidus*, and *Momordica charantia* extracts, may become promising alternatives to current drugs in diabetes treatment. This review provides keen insights into novel therapeutic strategies for the medical care of diabetes.

## Highlights

- The involvement of autophagy in the development of diabetes is corroborated by affecting the physiology and role of pancreatic β cells and the homeostasis of glucose.
- The prominent role of autophagy signaling pathway was supported by the alteration of autophagy markers in patients and animal models of T1DM, T2DM, and gestational diabetes.
- Several anti-diabetic strategies including adiponectin, ezetimibe, liraglutide, taurine, adipose tissue-derived stem cells (ADSCs) and even exercise as well as natural products such GABA tea, geniposide, guava extract, vitamin D have been shown to target autophagy.
- TGF-β, especially TGF-β1 as an uppermost isoform of TGF-β superfamily, may play a very essential role in the development of insulin resistance and obesity and finally, diabetes.
- Anti-diabetic drugs such as metformin and rosiglitazone have been reported to act *via* modulation of the TGF-β signaling pathway.
- Natural agents including compounds as resveratrol, puerarin, curcumin, hesperidin and silymarin, and extracts of propolis, *Lycopus lucidus*, and *Momordica charantia* have been shown to combat diabetes *via* modulation of TGF-β signaling pathway.

## Introduction

Diabetes mellitus (DM) is one of the most prevalent metabolic diseases worldwide. It is characterized by hyperglycemia and defective production and/or secretion of insulin and complications in the heart, kidney, and neural system leading to death, which have drawn notable attention to the management of diabetes. Among 451 million patients with diabetes, 5 million deaths were considered in the 20–99 years age range in 2017 (Cho et al., 2018). Despite recent achievements in the treatment of diabetes, it is important to continue deducing the molecular mechanisms of diabetes pathogenesis and shed light on new horizons for the complete treatment. Among several molecular mechanisms, autophagy and transforming growth factor–β (TGF-β) signaling pathways may play a causal role in the induction and progression of diabetes.

The involvement of dysregulated autophagy and TGF-β signaling pathways in the pathogenesis of diabetes and arising complications including cardiomyopathy, retinopathy, and nephropathy, has been reported in several studies (Jung et al., 2008; Levine and Kroemer, 2008; Al-Mulla et al., 2011; Bartolomé et al., 2014). The integrity of the autophagy pathway is a requisite to normal regulation of cells (Ebato et al., 2008). Autophagy induction is usually known as a protective mechanism to degrade unwanted components and proteins in cells (Klionsky and Emr, 2000). In the absence and/or dysregulation of autophagy, the accumulation of destructed proteins and components leads to deficits in cells. Activation of the TGF-β pathway is commonly associated with cell cycle arrest and induction of apoptosis, whereas altered signaling of TGF-β has been shown to play a substantial role in tumorigenesis (Zhao et al., 2018). However, one of the most conspicuous trends in recent years has been to evaluate the impact of TGF-β in the pathogenesis of other disorders such as diabetes (Wilson et al., 2017; Yadav et al., 2017). In addition, the potential cross-talk between autophagy and TGF-β has received a great deal of attention.

**Figure 1:**
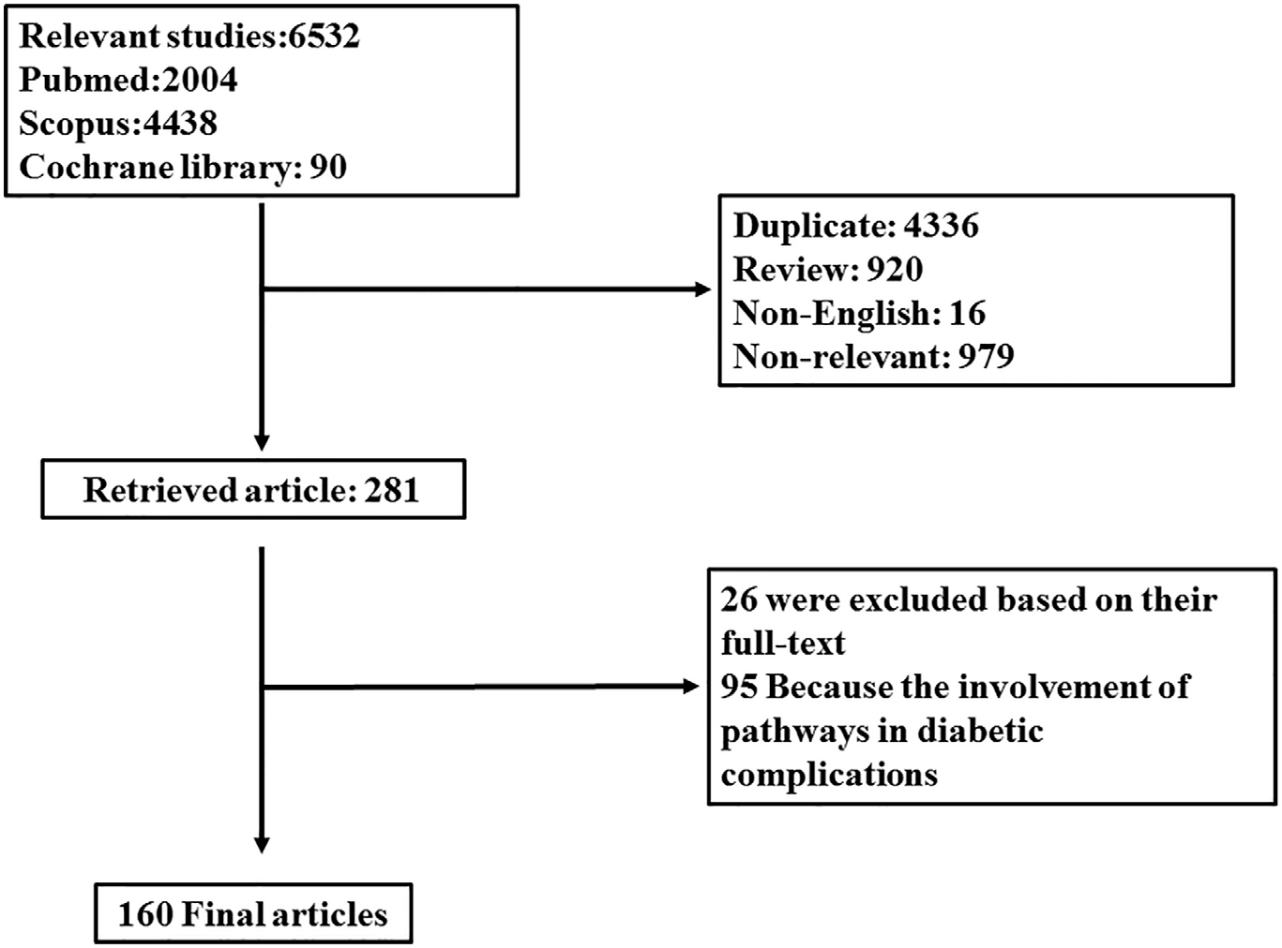
Flow diagram related to selection process of papers.

In light of this evidence, it is of interest to investigate studies regarding the involvement of autophagy and TGF-β signaling pathways and their cross-talk in the progression of diabetes to provide an impetus for identifying therapeutic strategies in the management of hyperglycemia and subsequent complications. For this aim, electronic databases including “Scopus,” “PubMed,” and “Cochrane Library” were searched using the keywords (“Autophagy” OR “mTORC” OR “LC3” OR “ATG” OR “TGFβ (TGF-beta)” OR “SMAD”) [all field] AND (“diabetes” OR “hyperglycemia”) [title/abstract/keyword]. Data was gathered from the inception date until January 2019. Among them, English language papers were solely included. Two independent investigators evaluated primarily obtained papers. From a total of 6,532 results, 4,336 papers were excluded because of duplication, 1,074 being irrelevant on the title and/or abstract, 920 reports for being reviews, and 16 because of language restriction. Among 281 retrieved papers, 26 were excluded according to their full text, and 95 were excluded because of the involvement of TGF-β and autophagy in diabetes complications including nephropathy, retinopathy, and neuropathy. One-hundred sixty articles were finally included in this systematic review. **Figure 1** discloses a flow chart of the study design.

## Biological and Pharmacological Aspects of Autophagy Signaling

Autophagy is a catch-all, self-eating process in which intracellular components are recruited to the lysosome for degradation. Macroautophagy, microautophagy, and chaperone-mediated autophagy, as the three main classes of autophagy, are involved in the lysosome-associated degradation of cytoplasmic constituents as well as unfolded proteins. Macroautophagy (hereafter known as autophagy) is an evolutionarily conserved mechanism characterized by double-membrane autophagosomes from cytoplasmic components that are fused with the lysosome to form an autophagolysosome for degradation of engulfed components with lysosomal enzymes (Klionsky and Emr, 2000). Consequently, an outstanding role of autophagy in multicellular organelles is the perseverance of homeostasis in metabolic, functional, and structural processes. While autophagy provides a driving force in the regulation of cell viability and function, it is also considered as the second type of programmed cell death in which the accumulation of autophagosomes distinguishes itself from apoptosis (Boya et al., 2005; Tsujimoto and Shimizu, 2005). Recent studies have contributed to a significant understanding of the cellular initiation and activation of autophagy signaling pathway (Hurley and Young, 2017; Yu et al., 2018). Specifically, the detailed molecular pathway behind the cellular function of autophagy and involved factors in mammals have recently been explored by a recent review article (Yu et al., 2018). As outlined in this review article, signaling factors such as ATG (AuTophaGy related proteins) family proteins, Unc-51 like autophagy activating kinase (ULK) complex, phosphatidyl inositol-3 phosphate (PI3P) and its producing complex, Ras-associated binding (Rab), 1A/1B-light chain 3 (LC3), 5′ adenosine monophosphate-activated protein kinase (AMPK), mitogen-activated protein kinase (MAPK), sulfiredoxin (SRX), WD-repeat protein interacting with phosphoinositides (WIPI), and soluble *N*-ethylmaleimide-sensitive factor-attachment protein receptor (SNARE) proteins implicated in the autophagy pathway (Yu et al., 2018).

**Figure 2:**
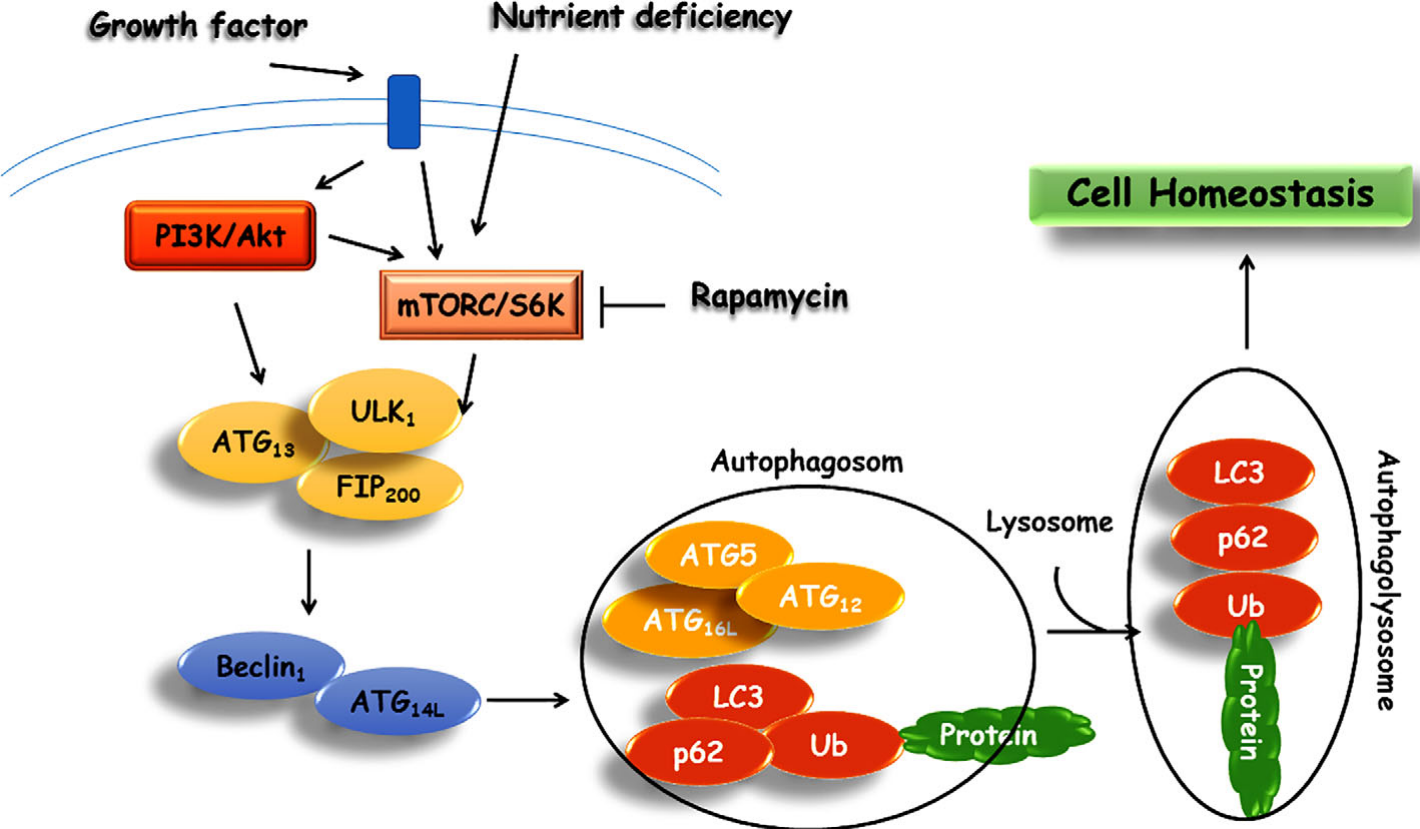
Schematic representation of autophagy signaling pathway.

The molecular and cellular mechanisms of autophagy are summarized in **Figure 2**. There are accumulating evidence emphasizing on the significance of autophagy’s role in the maintenance of cellular homeostasis (Eltschinger and Loewith, 2016). However, in spite of extensive studies over the past several years, the mechanism of autophagy remains only partially understood. It has been proposed that autophagy acts as a protective mechanism to retinue cells from the production of aggregative proteins and activation of inflammatory responses (Levine and Kroemer, 2008). The role of autophagy in metabolism management is supported by activation of the autophagy mechanism in a starvation state through inhibition of mTOR. Additionally, the impact of autophagy on metabolism was examined through the development of Atg7^+/-^ haploinsufficient mice in the normal condition where no disorders were observed. Yet, in crossed Atg7^+/-^ with ob/ob mice (deficiency of leptin), there were increased diabetogenic symptoms relative to ob/ob mice (Lim et al., 2014). From literature, dysfunctional autophagy leads to depreciated fat mass, increased degradation of hepatic lipid, and plunges in lipid levels in hepatocytes through activation of fibroblast growth factor 21 (FGF21), eventually leading to insulin resistance (Shibata et al., 2009; Singh et al., 2009; Zhang et al., 2009; Kim et al., 2013). The primary role of autophagy in the pathogenesis of diabetes is further corroborated by several studies on the physiology and function of pancreatic β cells (Kaniuk et al., 2007; Ebato et al., 2008).

### Role of Autophagy Signaling in Diabetes

Under physiological conditions, autophagy may be upregulated as a cellular defense mechanism. As mitochondria play a pivotal role in cellular endogenous ROS production and insulin biosynthesis and secretion, regulation of mitochondrial quality and quantity control through the selective engulfment of excessive or damaged mitochondria *via* autophagosomes promotes β cell health and is of paramount importance in preventing the progression of diabetes. In this line, an enhanced increase in mitochondrial oxidative stress and HbA1C levels resulted in mild hyperglycemia in patients with prediabetes, newly diagnosed type 2 diabetes mellitus (NDT2DM) and advanced duration of type 2 diabetes mellitus (ADT2DM). This increasing glycemic burden enhanced mitochondrial dysfunction in patients with T2DM. With the increased levels of oxidative stress, the aggregation of dysfunctional mitochondria was occurred in cells due to autophagy dysregulation (Scherz-Shouval and Elazar, 2007), resulted in increased insulin resistance, and acceleration T2DM disease progression (Petersen et al., 2003).

Examination of the classical autophagy markers, LC3II and LAMP2, revealed a remarkable rise in LC3-II and LAMP-2 mRNA expression in subjects with prediabetes (Rovira-Llopis et al., 2015), while patients with NDT2DM and ADT2DM showed the significant reduction in both LC3II and LAMP2 mRNA and protein expression. Mitochondrial autophagy (mitophagy) by clearing cells of damaged mitochondria *via* autophagosomes contributes to the improvement of prediabetic and diabetic symptoms. Mitophagy is regulated by several numbers of factors such as PTEN induced putative kinase 1 (PINK1), PARKIN, microtubule-associated protein light chain 3 (LC3), and lysosome-associated membrane protein2 (LAMP-2) and mitophagy receptors NIP3 like protein X (NIX) and mitofusin2 (MFN2) (Ding and Yin, 2012). Prediabetic subjects have been shown to possess an increased level of mitophagy biomarkers and mitochondria as compared to T2DM patients. It has also been demonstrated that the expression levels of Mitofusin-2 (MFN2), NIX, PINK1, and PARKIN were augmented in prediabetes in comparison with healthy ones. On a protein level, though NIX and PINK1 levels were comparable to the controls, MFN2 showed a significantly increased expression.

Among T2DM patients, a comparison between ADT2DM and NDT2DM showed a significantly decreased level of LAMP2 in patients with ADT2DM in comparison with NDT2DM, indicating the suppression of mitophagy induced by oxidative stress, resulting in further deterioration of survival. Moreover, a recent study by Møller et al. (2017) reported a decreased level of LC3II in muscle cells from T2DM patients as compared to the control subjects. Attenuation of LAMP-2 expression results in reduced autophagy, leading to β cell dysfunction and insulin resistance (Liu et al., 2013; Bhansali et al., 2017). In patients with NDT2DM and ADT2DM, researchers have been found a significantly reduced mRNA and protein expression of MFN2, NIX, PINK1, and PARKIN. In this line, oxidative stress-mediated by moderate to severe hyperglycemia was shown to be associated with a decreased level of these genes leading to impairment of mitophagy. Specifically, augmented oxidative stress in T2DM patients leads to impairment in PINK1 and PARKIN-mediated mitophagy, as delineated by the reduced content of LC3II and LAMP2 proteins, resulting in aggregation of disturbed mitochondria.

Binding of insulin to the insulin receptor (IR) is associated with phosphorylation of downstream targets including IRS-1 and IRS-2 (Withers and White, 2000) and subsequent activation of phosphatidylinositol 3-kinase (PI3K) signaling pathway, which is associated with activation of several processes such proliferation, cell growth, and glucose uptake. Suppression of insulin signaling pathway by ER stress is resulted in phosphorylation of IRS-1/2 by JNK and insulin resistance (Özcan et al., 2004). Preventing IR processing or reducing its expression has also been shown to cause insulin resistance and diabetes in humans (Yoshimasa et al., 1988; Formisano et al., 1993; Iwanishi et al., 1993). In 3T3-L1 adipocytes, it has been observed that ER stress through induction of autophagy results in down-regulation of the IR protein level (Özcan et al., 2004; Nakatani et al., 2005). This reduction in IR levels is accompanied by a decrease in IR downstream signaling and inducing insulin resistance in 3T3-L1 adipocytes. Specifically, the autophagy inhibitor 3-methyladenine (3-MA) utilization in 3T3L1 adipocytes has been shown to reveal the function of autophagy in triggering ER stress-induced IR degradation (Zhou et al., 2009). In addition, in 3T3-L1 adipocytes, glucose uptake under insulin stimulation is suppressed by ER stress suggesting that a reduced number of IR medicated by ER stress seems to play a causal role in insulin sensitivity (Zhou et al., 2009). However, normal glucose tolerance in IR (+/-) heterozygous knockout mice (Joshi et al., 1996), indicating that less IR may still exhibit expression in the range of normal. This is further complicated by research showing that inhibition of autophagy to ameliorate ER stress was not successful in 3T3L1 adipocytes; proposing that under stressful ER conditions, IR has been disturbed. Although, *in vitro* models of obesity, it has been demonstrated that chemical chaperones and/or overexpression of ER chaperone ORP150 may potentially alleviate insulin signaling and insulin sensitivity (Ozawa et al., 2005; Özcan et al., 2006).

Studies have documented that autophagy may act as an early event in experimental diabetes. streptozotocin (STZ)-induced diabetes leads to activation of VMP1-mediated autophagy in pancreatic β cells after 3 h administration (Grasso et al., 2009). The early detection of VMP1 and autophagic signals in STZ-treated rats and cells indicates that increased autophagy expression and related signals in β cells may play an important role as the recognized biomarkers of diabetes development. However, this supposition requires further evidence with future experiments (Grasso et al., 2009). Further validity was obtained through studies examining specific autophagic factors in mutagen and/or knockout animal models, namely, the Atg7-knockout mice (Atg7^Δβcell^) model (Jung et al., 2008; Fujitani et al., 2009). In one study, Ebato and colleagues clarified that the altered expression of autophagy factors, namely autophagy-related gene 7 (Atg7) knockout in mice fed a high-fat regimen, plays the causal regulatory role in maintaining the normal structure and function of β cells, leading to destruction of β cells and eventual insulin resistance (Ebato et al., 2008). In this line, dysregulated autophagy seems to play an important role in the pathogenesis of both T1DM and T2DM as well as their arising complications (Fierabracci, 2014). It is proposed that in β cells lacking autophagy, autoantigens are embarked on major histocompatibility complex (MHC) class I and are consequently recognized by T cell receptors on CD8^+^ T cells. This results in T cell activation and β cell destruction, where β and T lymphocytes are recruited to these inflammatory sites, such as pancreatic islets, with the production of autoantibodies (Lam-Tse et al., 2002). Development of the autoimmunity process leads to further activation of β and T cells, promoting huge lysis of β cells and insulitis. As a key to illuminate the crucial role of autophagy in diabetes progression, immunoblotting analysis unraveled the altered expression of some autophagic signals including light chain IIIB (LCIIIB), Beclin I, ATG12, and p62 proteins in non-obese diabetic (NOD) mice (Fierabracci, 2014).

Insulin resistance as a feature of T2DM is commonly associated with a progressive decrease in the β cell function and the emergence of hyperglycemia (Fujimoto, 2000; Kahn, 2000), mediating oxidative stress that hinders cell-reparative process like autophagy downstream. On the other hand, to maintain the β cell function and survival as well as insulin sensitivity at target sites, the involvement of autophagy seems to be crucial (Marchetti and Masini, 2009; Gonzalez et al., 2011; Kruse et al., 2015). It has been reported that decreased number of autophagosomes in β cells from ob/ob mice, implying the inhibition of autophagic degradation in insulin resistance (Abe et al., 2013). Furthermore, aggregation of polyubiquitinated proteins due to increased oxidative stress in the β cells of Zucker diabetic rats was shown to be mediated by autophagic dysfunction (Kaniuk et al., 2007). Moreover, a reduced level of IL-10 mRNA level has been reported in PBMCs from T2DM patients. Several studies have demonstrated that mTOR activation has a direct relationship with the expression of IL-1β and TNF-α but has a negative relationship with IL-10 through the IKKβ (Laplante and Sabatini, 2012; Shigihara et al., 2014). Notably, evaluation of the link between inflammation and autophagy revealed that autophagy play the main role in regulating inflammation (Ma and Blenis, 2009; Menzies et al., 2011) in macrophages (Kaushik and Cuervo, 2012), keratinocytes (Menon et al., 2014), hypothalamus (Hardie, 2003), adipocytes (Rivera et al., 2014), and peripheral blood mononuclear cells (PBMCs) (Lempiäinen and Halazonetis, 2009). In PBMCs of T2DM and non-diabetic (ND) subjects, mRNA expression of BECN1, LAMP2, and LC3B decreased as the protein level of p62/SQSTM1 was increased (Inoki et al., 2003), meaning that the autophagic process decreased. The decline in protein levels of LC3B-II is accompanied by the rise in TNF-α and IL-1β expressions, while the increase in the protein level of p62 parallels the reduction in them. Thus, It seems that decreased autophagy in PBMCs of T2DM may be due to hyperglycemia (Egan et al., 2011; Guillén and Benito, 2018). However, the inhibitory effect of mTOR on autophagy pathway may be another motive (Sancak et al., 2007; García-Aguilar et al., 2016) as its activation attenuates the Beclin 1 gene expression in PBMCs. AMPK also plays the premier role in the regulation of autophagy (García-Aguilar et al., 2016), albeit with inconspicuous involvement relative to mTOR (Guillén and Benito, 2018).

It is a fact that T2DM could be characterized by chronic inflammation in which leukocytes are impaired in function (Hernandez-Mijares et al., 2013; Rovira-Llopis et al., 2014). Hyperglycemia-induced oxidative stress and ER stress are well-known mechanisms involved in the progression of T2DM (Gonzalez et al., 2011; Scherz-Shouval and Elazar, 2011). A large cohort study indicated that autophagy is enhanced in the leukocytes of T2DM patients. It has been shown that regulation of autophagy in diabetes is different depending on cell types, namely, autophagy is activated in the pancreatic β cells and inhibited in the liver of T2DM mice. In the leukocyte of T2DM patients, an increase in autophagy marker, Beclin 1, is in accordance with the increase in intracellular ROS level (Rovira-Llopis et al., 2015). Although autophagy should control this ROS accumulation by removal of damaged mitochondria, leukocytes from T2DM patients indicate that autophagy activation is not sufficient to reduce ROS production (Hubbard et al., 2010). In addition to oxidative stress, ER stress upregulates autophagy *via* inhibition of the AKT/TSC/mTOR pathway by mediating LC3 lipidation to LC3-II in conjunction with the activity of PERK/eIF2a phosphorylation and by transcriptional activation of autophagy-related genes (Gonzalez et al., 2011).

**Figure 3:**
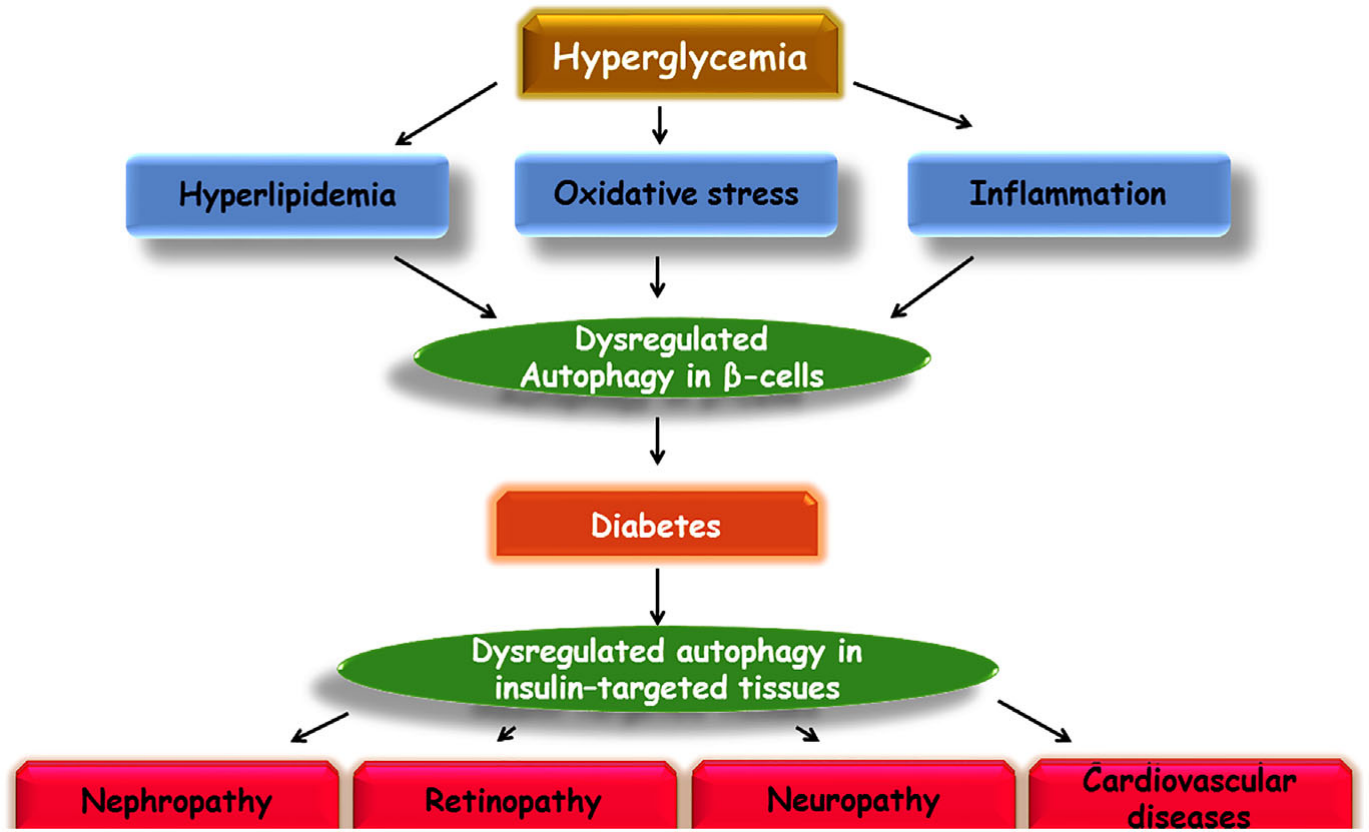
Schematic illustration of a proposed model of autophagy involvement in diabetes and its complications.

In several *in vitro* and *in vivo* studies to evaluate the role of an amyloidogenic protein in β cells, human islet amyloid polypeptide (hIAPP) was shown to play the causal role in the pathogenesis of T2DM. Furthermore, a reciprocal interplay was found between the clearance of hIAPP and the activation of autophagy, where autophagy deregulation led to failed clearance of hIAPP and subsequently aggregated hIAPP is associated with decreased activity of the autophagy system (Kahn et al., 1999; Morita et al., 2011; Rivera et al., 2011; Shigihara et al., 2014). Neuronal dysfunction and cognitive impairment in dementia have also been shown to have a strong association with β cell failure, such that AD is known as the type 3 diabetes in literature (Ott et al., 1996; Steen et al., 2005; Moroz et al., 2008; Chen and Zhong, 2013). The role of autophagy in the development of memory dysfunction was scrutinized in STZ-induced diabetic rats, where expression of Aβ_1-42_ and autophagic markers, LCII and beclin 1, was increased in the hippocampus. Adversely, the reduced expression of lysosome factors, such as LAMP1 and LAMP2, illustrated the involvement of lysosome dysfunction in the neurodegenerative impact of diabetes. These results endorse the impact of autophagy and lysosome deregulation in neuronal loss in diabetic conditions (Ma et al., 2017). As another example, it was shown that high fat-fed, STZ-induced diabetic mice are vulnerable to cognitive dysfunction through altered expression of autophagy signaling factors including LCII/I, p62, and beclin1 (Guan et al., 2016) (**Figure 3**).

### Drugs Targeting Autophagy Signaling for Therapeutic Benefits in Diabetes

#### Adiponectin

Adiponectin is a metabolic hormone secreted by adipose tissue. It has been shown that adiponectin acts as a diabetes regulating hormone (Cheng et al., 2014) that activates the autophagy pathway in insulin target cells and decreases in metabolic diseases. Adiponectin mediates direct metabolic effects and improves insulin sensitivity by the autophagic cellular mechanism (Jahng et al., 2015; Xu and Sweeney, 2015). For example, the mechanism of adiponectin demonstrated the involvement of the AMPK signaling pathway in improving insulin sensitivity and glucose tolerance in db/db mice or mice fed a high-fat diet (Okada-Iwabu et al., 2013). Moreover, insulin resistance in L6 skeletal muscle cells (Huang et al., 2002b) revealed that the increase in expression of a GRP78 promoter-dependent fluorescent reporter and IRE1 phosphorylation (pIRE1), pPERK, and ATF6 is induced under treatment of high insulin/glucose (HIHG) and is paralleled by ER-stress induction (Ahlstrom et al., 2017), while adiponectin treatment alleviated ER stress in an autophagy-dependent manner *via* activation of AMPK signaling factor.

#### Ezetimibe

Mounting evidence indicates a direct relationship between metabolic disorders, such as obesity, liver diseases and insulin resistance (Clark, 2006; Byrne, 2012). Owing to the correlation between abnormal cholesterol metabolism and the development of metabolic diseases, it appears that the same drugs could be beneficial for both types of diseases. Ezetimibe treatment is a good example of both decreasing intestinal cholesterol incorporation by blocking Niemann-Pick C1-like (NPC1L1) protein (Altmann et al., 2004; Garcia-Calvo et al., 2005) and as described in previous studies, improving glycemic control, leading to an increase in bioactive GLP-1 and pancreatic β cell mass in Otsuka Long-Evans Tokushima Fatty (OLETF) rats (Yang et al., 2011). Furthermore, the expression of ATG5, ATG6, and ATG7 in the liver was considerably increased in ezetimibe-treated OLETF rats (Chang et al., 2015), demonstrating the efficacy of ezetimibe treatment in improving impaired autophagy process and increased ER stress in insulin resistance disease.

#### Liraglutide

Liraglutide is a synthetic peptide with 97% sequence homology to native human GLP-1 and is a promising anti-diabetic dug (Drucker and Nauck, 2006; Lovshin and Drucker, 2009). Injection of liraglutide, which can cross the blood-brain barrier, may prove as a neuroprotective agent in animal models with neurologic disorders, such as cerebral ischemia (Zhu et al., 2016), traumatic brain injury (Li Y. et al., 2015), stroke (Sato et al., 2013), and Alzheimer’s complications (Han et al., 2013). As a result, liraglutide may attenuate DM-induced cognitive decline in mice.

Cognitive impairments, DM-induced hippocampal neuronal injuries, and synaptic ultrastructure degradation were shown to decrease in STZ-induced diabetic mice under liraglutide treatment, which recruits the AMPK/mTOR pathway to elevate the autophagic process (Qi et al., 2016). Additionally, improvements in lesions in neuronal morphology and density in the hippocampal CA1 region have also been observed after chronic liraglutide administration. It has been suggested that liraglutide did not have protective effects in STZ-induced diabetic mice. Similarly, earlier data from preclinical and clinical studies also indicated that GLP1 and its analogues had no significant improvement in the glucose levels or body weight (Zhao et al., 2013; Frandsen et al., 2015; Dejgaard et al., 2016; Dietrich et al., 2016; Hernández et al., 2016; Zanotto et al., 2017).

#### Taurine

Taurine is a free amino acid and natural compound that shows promising results for improving impaired glucose metabolism by enhancing the low levels of PPARγ and mTORC2 expression induced by inorganic arsenic (iAs) in the liver of mice and HepG2 cell line. In fact, taurine administration may ameliorate iAs‐induced insulin resistance through activation of PPARγ‐mTORC2 signaling and subsequent inhibition of hepatic autophagy. Although autophagy activation contributed to the relief of insulin resistance with treatment by various compounds (Shi et al., 2015; Li et al., 2017), taurine effectiveness in insulin resistance is obtainable through autophagy inhibition.

### Natural Products Targeting Autophagy Signaling in Diabetes

#### GABA Tea

Several mechanisms including decreased γ-aminobutyric acid (GABA) neurotransmission, oxidative stress, and apoptosis have been considered for the pathogenesis of encephalopathy in diabetes. Therefore, several studies have carried out experiments on the effects on diabetic encephalopathy through the administration of GABA tea (Hininger-Favier et al., 2009; Zhao et al., 2011). Reduced GABA uptake (Duarte et al., 2000) and extracellular GABA were reported in hyperglycemia, which attributed to increased neuronal disorders. Consequently, it was observed that the administration of GABA tea with enriching GABA neurotransmitters in diabetic animals exerts hypoglycemic and anti-apoptotic effects on rat brain cerebral cortex.

GABA tea also known as Gabaron, developed for the first time in Japan, is a new form of tea in which during a fermentation process, GABA is accumulated in the leaves of tea. The common use of GABA tea is in the amelioration of blood pressure (Omori et al., 1987; Abe et al., 1995). Further examination of the mechanism whereby GABA tea ameliorates the neurodegenerative symptoms of diabetes in the brain of STZ-induced DM rats revealed that blood glucose levels increase in STZ-induced diabetes rats and improvement of hyperglycemia is achieved by deactivation of the cortical Fas ligand, Fas-associated death domain protein (FADD), caspase-8, Bid, and t-Bid levels—all of which increased following 4 weeks of STZ-induced diabetes. Moreover, signaling factors, such as Bax, cytochrome c, activated caspase 9, and activated caspase 3 in the cerebral cortex of STZ-induced diabetes rats, were significantly increased compared with non-diabetic rats. In addition, GABA tea exposure was associated with suppression of the apoptotic pathways mediated by diabetes. Another cellular process affected by GABA tea in STZ-induced diabetes is autophagy as seen by decreases in related protein levels including Beclin 1, ATG7, ATG12, LC3-I, and LC3-II following treatment with GABA tea.

#### Geniposide

One potential drug for the regulation of abnormal signaling pathways in diabetes is geniposide, an iridoid compound isolated from *Gardenia jasminoides* J.Ellis with anti-inflammatory, anti-angiogenesis, and anti-tumor activities (Lee et al., 1995; Koo et al., 2004; Koo et al., 2006). Furthermore, it is significantly capable of promoting glucose uptake (Guo et al., 2012). When HepG2 cells in IR were treated with 62.5 mg/L geniposide, the decreased levels of glucose were shown in the supernatant in a time‑dependent manner. Consequently, it is thought that geniposide promotes autophagy in insulin resistance HepG2 cells, leading to the inhibition of NF‑κB signaling factor and reversing the inhibitory impact of NF‑κB on the expression of GLUT‑4, thereby increasing mRNA and protein expression levels of GLUT‑4. As such, geniposide may aid in insulin resistance treatment.

#### Guava Extract

Guava, *Psidium guajava* L. (Myrtaceae), is a tropical fruit with anti-oxidative, anti-inflammatory, and anti-diabetic attributes (Li P. Y. et al., 2015) because of an increased level of vitamin C, flavonoids, and polyphenolic ingredients (Flores et al., 2013). In particular, the effectiveness of guava extracts in the reduction of ROS, protection against inflammation in the kidney, DM-induced sclerotic injury, and cell arrangement in the pancreas has been observed (Lin and Yin, 2012). Moreover, the impact of guava leaf extracts/trehalose treatment on T2DM was confirmed in some studies (Eidenberger et al., 2013; Lin et al., 2016). Trehalose as a disaccharide found in almost all of the organisms has various therapeutic effects on T2DM (Bartolomé et al., 2010). With this in mind, the effect of trehalose in combination with guava extract was observed as a potent scavenger of intracellular ROS in STZ-induced diabetic mice. Along with T2DM-enhanced renal ROS, three types of programmed cell death including apoptosis, autophagy, and pyroptosis were significantly diminished using guava juice and trehalose exposure. While autophagy in diabetic patients has mainly been proved to be a protective process against ER-stress, guava juice and trehalose affect DM positively by reducing autophagy leading to cell death (Lin et al., 2016).

#### Vitamin D

There is the enormous evidence for considering vitamin D deficiency as one of the main causes of T1DM development (Wolden-Kirk et al., 2011; Wranicz and Szostak-Wegierek, 2014; Grant, 2015). The protective role of vitamin D against diabetes has been suggested in several studies (Sørensen et al., 2012; Dong et al., 2013). For example, pre‑treatment with 1,25(OH)_2_D3 promoted insulin secretion in STZ‑treated β cells, confirming this notion. Examination of the precise underlying mechanisms of vitamin D in STZ‑induced T1DM mouse model and mouse insulinoma 6 (MIN6) β cells revealed that vitamin D increases the expression of LC3 and Beclin 1, autophagic signaling factors that may influence the promotion and development of diabetes (Lee, 2014; Ding and Choi, 2015). Furthermore, enhanced expression of Bcl‑2 in STZ‑treated mouse and MIN6 cells may serve as a good indicator for showing the decrease in the apoptotic rate of pancreatic β cells. Indeed, vitamin D may induce autophagy and suppresses apoptosis, therefore accelerating the regeneration of organelles under hyperglycemic conditions (Wang et al., 2016).

## Biological and Pharmacological Aspects of TGF-Beta Signaling

Transforming growth factor-beta (TGF-β) was identified during the early 1980s. Subsequently, TGF-β signaling was elucidated in the middle of the 1990s with the identification of SMAD proteins and TGF-β receptors. Since the first identification of TGF-β, research is in progress to expound the TGF-β signaling pathway and its pathophysiological importance. TGF-β signaling has been studied in detail at the cellular and molecular level by various scientists. Particularly, numerous cross-talks have emerged regarding the relationship of TGF-β with other pathways as well as the role of the TGF-β signaling pathway in the development of various diseases. TGF-β is grouped in a family of peptide growth factors that are involved in the developmental processes, homeostasis of adult tissues, and a wide range of other cellular functions. The impairment in TGF-β signaling has been reported in several diseases including cancer, diabetes, and cardiovascular diseases. The basic components of the TGF-β signaling consist of a receptor complex with membrane-associated receptors type I and type II and SMAD proteins, which play a role in downstream transcriptional factors. Type I and type II TGF-β receptors are similar in composition. The receptors are composed of a cytoplasmic kinase domain, a single transmembrane segment, and an ectodomain that is glycosylated and disulfide-rich. Serine/threonine kinase activity and induced phosphorylation of tyrosine are characteristic features of kinase domains in TGF-β receptors. There are seven different types of type I TGF-β receptors, which are referred to as activin receptor-like kinases (ALKs). Specifically, ALK5 or TGF-βRI is responsible for TGF-β signaling in all types of mammalian cells. For example, ALK5 works alongside ALK1 and ALK2 in endothelial or other various cell types (Yan and Zhang, 2018; Zhang, 2018).

**Figure 4:**
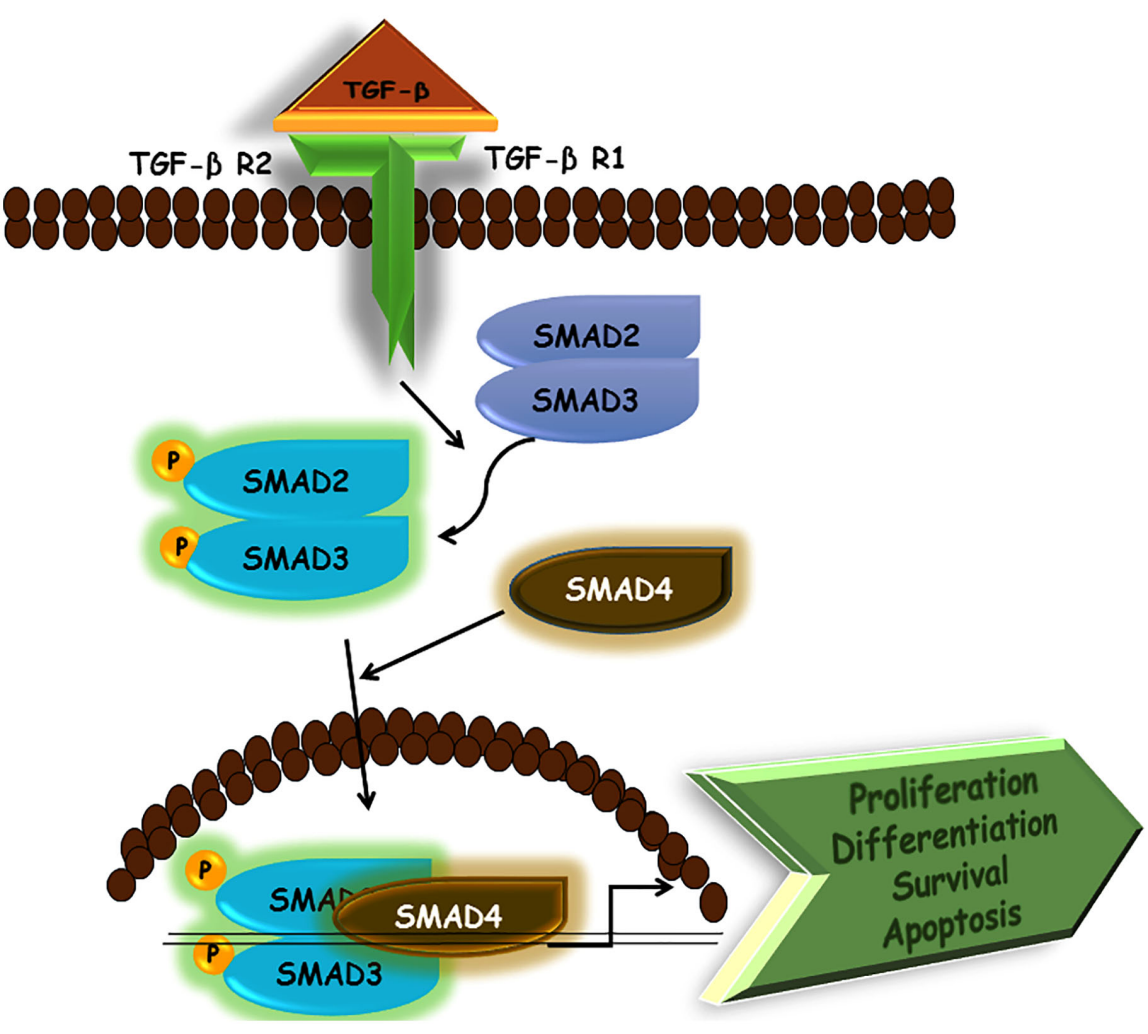
Schematic representation of TGF-β signaling pathway.

In TGF-β signaling, ligand binding allows the receptor complexes to assemble and activated type II TGF-β receptors may phosphorylate type I receptor’s glycine-serine-rich (GS) domain. This leads to the activation of type I receptors. Then, the activated type I receptors phosphorylate the SMADs at their carboxyl terminus, aiding SMADs to enter the nucleus to regulate gene expression. Different SMADs are encoded in the mammalian genomes and possess different features. Examples include SMAD 2/3 for ALK5 and SMAD 1/5/8 for ALK1/2, which act as direct substrates for their cognate receptor kinases. By virtue of this ability, they are also referred to as receptor-specific, or R-SMADs. Further downstream signal transducers, such as small guanosine triphosphatases (GTPases), MAPKs, Janus kinase/signal transducer and activator of transcription (JAK/STAT), and phosphoinositide 3-kinase/protein kinase B (PI3K/AKT) are also activated by TGF-β receptors. When activated, these various signal transducer pathways perform specific functions, such as converging to SMADs to influence the output of TGF-β signaling. Thus, TGF-β signaling not only has its specific role in the body but influences other pathways as well; thereby explaining why dysregulation in TGF-β signaling is involved in several diseases. Non-SMAD signaling transducers can also be activated by TGF-β. One of the most important non-SMAD effectors is ubiquitin E3 ligase tumor necrosis factor receptor-associated factor 6 (TRAF6). TRAF6 produces activation of downstream p38 MAPK, c-Jun N-terminal kinase (JNK), and transforming growth factor beta-activated kinase 1 (TAK1) (Yan and Zhang, 2018; Zhang, 2018). As such, non-SMAD pathways may also shed light on the variety of proteins that TGF-β receptors interact with them. This serves as a greater option for cells to influence downstream responses in accordance with pathological and physiological demands (**Figure 4**).

## Role Of Tgf-β Signaling In Diabetes

**Figure 5:**
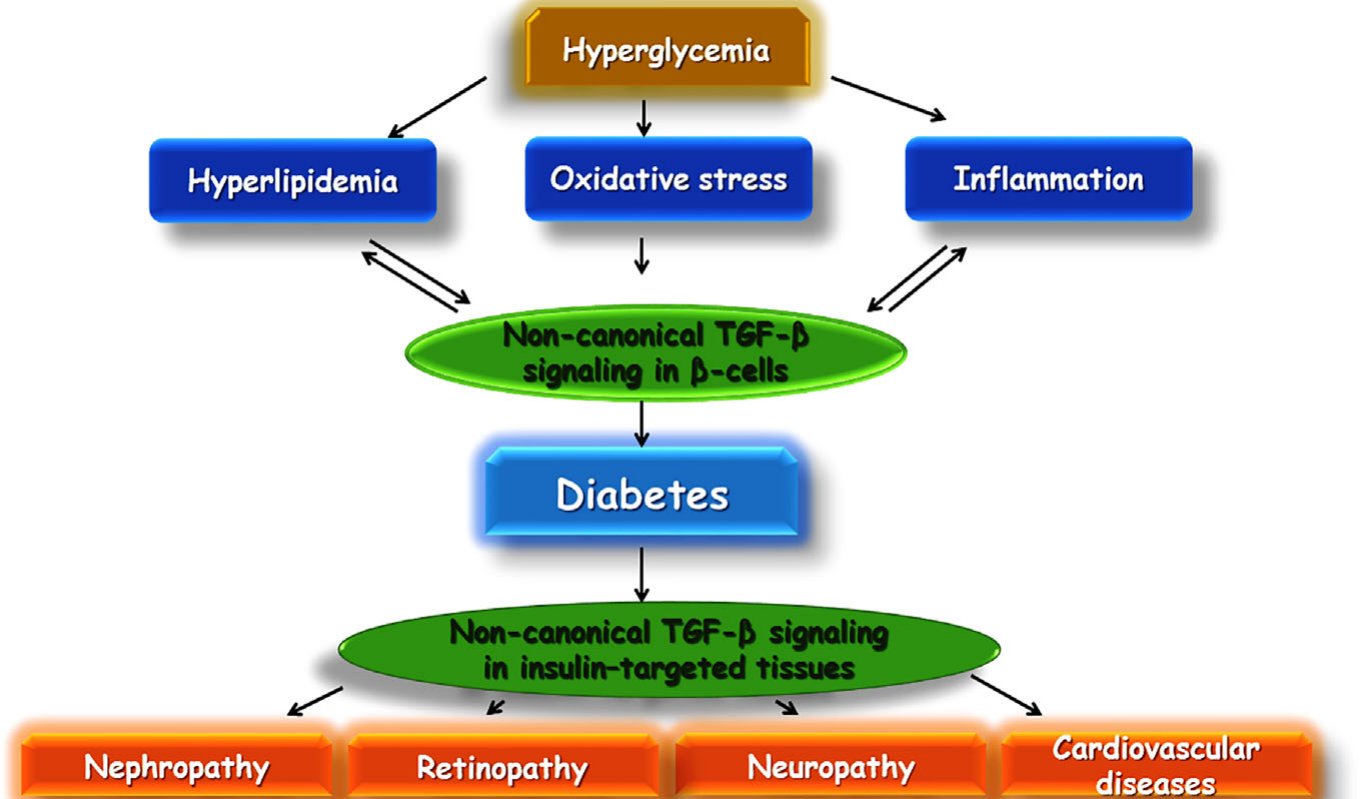
Schematic illustration of a proposed model of TGF-β involvement in diabetes and its complications.

TGF-β is a cytokine with several numbers of functions inside the body consisting of apoptosis, immune response in several cells, differentiation, cell proliferation, and wound healing. Recent experimental research has pointed out that TGF-β may play a very essential role in the development of insulin resistance and obesity (Beaudoin et al., 2014). There is evidence that TGF-β1 (a predominant isoform of the superfamily of TGF-β) may be involved in the development of diabetes (Flores et al., 2004). Additionally, TGF-β plays an important role in the immune system. It is involved with differentiation, chemotaxis, survival, and lymphocyte proliferation. Regulation of leukocyte function is an important role played by TGF-β, and dysregulation can lead to autoimmune diseases, such as type T1DM. In T1DM, the destruction of insulin-producing β cells takes place and is mediated by T cells, important targets of TGF-β1. Blocking of TGF-β signaling in mice has led to an autoimmune phenotype, involving activation and differentiation of T cells. In β cells, TGF-β1 is expressed under the influence of insulin promoters and inhibits T1DM from developing (Chen et al., 2008) (**Figure 5**).

A study using human subjects evaluated changes in the levels of TGF-β2 and nerve growth factor in T1DM patients compared with normal subjects and T2DM patients. It was found that levels of TGF-β2 were significantly lower and levels of nerve growth factor were higher in T1DM patients when compared to healthy controls and T2DM patients (Azar et al., 1999). In another study, TGF-β1 levels were evaluated in women with a previous history of gestational diabetes mellitus (GDM) due to increased risk of insulin resistance, obesity and endothelial dysfunction later in life and early development of atherosclerosis. Therefore, this study consisted of women with a prior history of GDM (pGDM), women with T2DM, and the third group of healthy women. The results showed that women with pGDM had significantly higher levels of TGF-β1 than healthy women, but lower levels of TGF-β1 than the T2DM group. The study indicated that age, postprandial glucose levels, and BMI all affected TGF-β1 levels. The elevation in TGF-β1 levels may be a result of the inflammatory response that is produced against insulin resistance and hyperglycemia (Yener et al., 2007). In another study carried out to study oxidation, glycation, and TGF-β1 levels, TGF-β1 levels were measured in children suffering from type I diabetes mellitus and healthy children. It was observed that parameters related to oxidation and glycation were considerably augmented in diabetic children compared to healthy children, such as TGF-β1 levels. Furthermore, correlation existed between the TGF-β1 levels and the age of the children and the duration of type I diabetes mellitus. However, no correlation existed between parameters for oxidation and glycation and levels of TGF-β1 (Jakuš et al., 2012).

Dysregulation of TGF-β pathway is specially associated with progression of various complications associated with DM, such as diabetic neuropathy, and delayed wound healing. Features of diabetic neuropathy include glomerular sclerosis, tubulointerstitial fibrosis, extracellular matrix (ECM) alterations, and mesangial expansion. TGF-β1 is an important regulator of fibrosis associated with diabetic nephropathy as indicated by increased renal TGF-β1 expression in persistent hyperglycemic conditions of human patients and animal models of diabetes. The effects of TGF-β1 are produced as a result of binding to TGF-β1 type II receptors (TβRII) and subsequently, induces activation of TGF-β1 type I receptor (TβRI) kinase. Consequently, this results in phosphorylation and activation of SMAD 2/3. Oligomeric complexes of activated SMAD2/3 are then formed with SMAD4 and translocated to the nucleus, leading to the expression of target genes, such as extracellular matrix (ECM) proteins as well as the production of tubulointerstitial and glomerular fibrosis. Thus, diabetic renal fibrosis may be treated by inhibiting the TGF-β1/SMAD pathway (Wu et al., 2015).

The deregulated vascular system in the eyes and kidneys from T1DM patients has been suggested to be affected by several factors including the duration of diabetes and deregulation of several signaling factors such as vascular endothelial growth factor (VEGF), TGF-*β*1, and angiogenin (Zorena et al., 2009). Further, TGF-*β*1 participation in vasculature and wound healing has been proved *via* the promotion of extracellular matrix proteins formation (Maheshwari et al., 2011). The measurement of TGF-*β*1 serum levels in children and adolescents revealed a positive relationship between the duration of T1DM and complications in the vascular system (Zorena et al., 2013). Augmentation of pro-inflammatory cytokines secreted by the peripheral blood mononuclear cells (PBMCs) is associated with atherosclerotic damages in T2DM (Ebato et al., 2008; Zoncu et al., 2011; Lin et al., 2012) and results in resistance to insulin and abnormality in β cell.

TGF-β serves as an important regulator of wound healing as it is released by platelets at an early stage and plays many roles downstream. For example, TGF-β is involved with chemotaxis of immune and inflammatory cells to the wound site and formation of granulation tissue and deposition of ECM. TGF-β also plays an important role in the end stages of tissue remodeling in wound healing by aiding replacement of collagen type III with collagen type I. TGF-β also plays an important role in wound healing by promoting epithelialization of wound. Thus, therapeutic benefits may be achieved in diabetes by either producing inhibitory or stimulatory effects on the TGF-β pathway depending on what effect is needed (Hozzein et al., 2015). An animal study was conducted to study the defective resolution of inflammation and impaired TGF-β signaling in delayed wound healing in a female rat model of T2DM. This clarified that wound healing was delayed due to elevated tumor necrosis factor (TNF)-α/NF-κB activity and decreased estrogen levels, which leads to decreased TGF-β/SMAD signaling and impaired inflammation resolution. However, PEGylated soluble tumor necrosis factor receptor type 1 (PEG-sTNF-RI) therapy and estrogen treatment produced amelioration in the above-mentioned defects (Al-Mulla et al., 2011).

A study was carried out to measure urinary TGF-β1 levels in patients suffering from DM, due to the role of TGF-β1 in enhancing renal fibrosis in diabetic neuropathy (DN). In a study, urinary levels of TGF-β1 were measured in groups consisting of healthy controls and patients suffering from DN. The levels of TGF-β1 were found to be higher in diabetic patients suffering from diabetic neuropathy compared to those belonging to the normal control group (Tsapenko et al., 2013). Another study on the role of TGF-β in DN utilized subjects that were grouped into those who had a fast development of DN, those with the slow development of DN, and healthy humans serving as the control. In this study, cultured skin fibroblasts of the subjects were evaluated for messenger ribonucleic acid (mRNA) expression levels for latent TGF-β binding protein-1 (LTBP-1), thrombospondin-1, TGF-β type II receptor (TGF-β RII), and TGF-β1. Measurements were collected using real-time RT-PCR. The results concluded that mRNA expression of LTBP-1 was reduced in patients belonging to the slow development of DN compared to both patients with the fast development of DN and control subjects. Additionally, thrombospondin-1, TGF-β RII, and TGF-β1 mRNA expressions were found to be similar in all the groups. Low levels of LTBP-1 may point towards a genetically determined protective effect against DN. The study also suggests that LTBP-1 may be involved in the development of DN *via* regulation of TGF-β activity (Huang et al., 2002a).

### Drugs Targeting TGF-Beta Signaling for Therapeutic Benefits in Diabetes

Drugs that target the TGF-β signaling may be potential candidates for therapeutic benefits in DM. Among them, rosiglitazone is conventionally used as an agonist for the proliferator activated receptor-gamma (PPAR-γ) for the treatment of DM. In a recent study, the effects of rosiglitazone were studied on the TGF-β/SMAD signaling pathway in Zucker diabetic fatty (ZDF) male rats. One group of rats received a chow diet and rosiglitazone treatment while the other group received chow diet without rosiglitazone treatment. The treatments were given for the duration of six weeks and rosiglitazone was administered in a dose of 100 mg/kg. Excision of retroperitoneal white adipose tissues (rpWAT) and subcutaneous white adipose tissues (scWAT) was performed to evaluate protein content/phosphorylation. It was found that in both scWAT and rpWAT, the protein content of mitochondria and glucose tolerance was found to be increased. However, the protein content of fatty acid handling enzymes was only shown to be elevated in the scWAT of animals that received rosiglitazone. Specifically, there was an elevation in the expression of SMAD4, TGF-β receptor I and II, and anchor of SMAD for activation of the receptor. Additionally, administration of rosiglitazone elevated levels of E3 ubiquitin ligase SMURF2 and inhibitory SMAD7 as well as reduced the phosphorylation of SMAD2 and SMAD3. These results indicate that rosiglitazone specifically inhibits signaling produced by SMAD2 and SMAD3 in scWAT. Besides, the SMAD7 and SMURF2 mechanisms induced by rosiglitazone are most likely responsible for decreasing phosphorylation of SMAD2 and SMAD3. A feedback mechanism is formed by activation of the SMAD signaling factors to oppose rosiglitazone induced synthesis of lipid in scWAT (Beaudoin et al., 2014).

Metformin has also been used as an antidiabetic agent for a long time. However, the exact mechanism of metformin is not clear. Yet, certain researchers have found that TGF-β1 may be a target for the action of metformin. A surface plasmon resonance-based assay was used by researchers to explore the effect of metformin on TGF-β1. It was found that metformin showed direct binding with TGF-β1 and inhibited binding of TGF-β1 with its receptor. Binding of TGF-β1 with metformin at the receptor-binding domain of metformin was demonstrated in the molecular dynamic and molecular docking studies. Additionally, metformin suppresses the dimerization of type II TGF-β1 receptor upon binding TGF-β1, which is essential for downstream signal transduction in the TGF-β pathway (Xiao et al., 2016). In a study on diabetic rats, it was shown that vitamin D produced improvement in TGF-β and insulin like growth factor 1 (IGF-1) levels in their intervertebral disc. However, the administration of vitamin D (calcitriol) had a protective effect against degenerative changes produced in the intervertebral disc of diabetic rats and improved IGF-1 and TGF-β levels. Thus, by increasing the levels of TGF-β1 and IGF-1, vitamin D may be helpful in the prevention and treatment of intervertebral disc degeneration in patients suffering from diabetes (An et al., 2017). A study was conducted to observe the effect of undenatured camel whey protein in enhancing wound healing in diabetic mice. Researchers found that whey protein increased the expression of TGF-β, fractalkine (CX3CL1), KC (keratinocyte-derived chemokine), MIP (macrophage inflammatory proteins)-2, and MIP-1α levels in diabetic mice treated with whey protein. Additionally, levels of IL (interleukin)-6, IL-10, TNF-α, and IL-1β were restored to normal levels through whey protein treatment. Thus, the actions of whey protein exerted a beneficial effect on wound closure in diabetic mice (Badr et al., 2012).

### Natural Products Targeting TGF-β Signaling in Diabetes

Natural products may be used for targeting TGF-β signaling and inducing therapeutic benefits in diabetes. The advantage of these natural products is that they may be safer alternatives in the treatment of diabetes in comparison to allopathic drugs. By targeting TGF-β signaling, they could play a novel role in the treatment of diabetes in comparison to standard drugs. Some of the natural products that may be used in the targeting of TGF-β signaling for therapeutic benefits in diabetes are as follows:

#### Resveratrol

Resveratrol (3, 5, 4′-trihydroxystilbene) is a phenolic compound with very important health benefits (Yeung et al., 2019). Resveratrol is found in several plants and is effective in the treatment of a number of age-dependent, metabolic and chronic diseases such as cancer, Alzheimer’s, diabetes, inflammation, bacterial and viral infections (Pawar et al., 2017; Moosavi et al., 2018). In a study, resveratrol improved diabetic neuropathy in STZ-induced diabetic rats. Resveratrol exhibited an effect on diabetic neuropathy by inhibiting the TGF-β/SMAD and extracellular signal-regulated kinase (ERK)1/2 signaling. Streptozotocin was administered in a dose of 65 mg/kg body weight. Induction of diabetes was confirmed by diabetic symptoms in rats, such as polyphagia, polydipsia, and fasting blood glucose of ≥300 mg/dL. Resveratrol was administered to animals at a dose of 0.75 mg/kg body weight for 8 weeks and three times per day. Animals were divided into groups in the following manner: normal animals receiving only normal saline, diabetes rats administered resveratrol treatment, and diabetic rats not administered resveratrol treatment. After the test, animals were sacrificed and histology of their kidney was examined by using microscopy. Biochemical and other important parameters were measured to evaluate the effect of resveratrol in diabetic rats. It was shown that glomerular hypertrophy and urinary albumin excretions as well as, expression of collagen-IV, fibronectin, and TGF-β in the glomeruli, were reduced in rats that received resveratrol treatment. Specifically, the thickness of the glomerular basement membrane was reduced to original thickness *via* resveratrol treatments while the expression of nephrin was enhanced to normal levels in diabetic rats administered resveratrol. In the kidneys of diabetic rats, phosphorylation of SMAD2, SMAD3, and ERK1/2 was shown to be inhibited by resveratrol. Therefore, this study suggests that resveratrol reduces early glomerulosclerosis in diabetic nephropathy through inhibition of ERK1/2 and TGF-β/SMAD. Furthermore, resveratrol reduces podocyte injuries in diabetic rats (Chen et al., 2011).

#### *Lycopus lucidus* Turcz. ex Benth.

*Lycopus lucidus* Turcz. ex Benth. is a medicinal plant used in Chinese herbal medicine. It has a traditional phytomedicine with anti-inflammatory, antioxidant, antimicrobial, anti-allergic, anti-osteoclastogenesis, anti-cancer, and anti-diabetic properties (Shin et al., 2005; Yu et al., 2011; Yao et al., 2013; Lu et al., 2015; Jeong et al., 2019). The aqueous extract of *L. lucidus* was used in a study to improve renal damage in STZ-induced diabetic rats with diabetic nephropathy. Two models were used in this study for determining renal fibrosis: an *in vivo* model in which STZ was used for inducing diabetic nephropathy in rats and an *in vitro* model where renal fibrosis was determined by treating fibroblasts with recombinant TGF-β1 (rhTGF-β1). Results showed that the aqueous extract of *L. lucidus* suppressed the activation of ERK1/2 and SMAD2 by rh-TGF-β1. The aforementioned effect also downregulated the expression of SMAD7, SMAD4, TGF-βRII, and TGF-βRI in SV40MES13 cells without any inhibitory effect on cell viability. In the *in vivo* rat model, *L. lucidus* reduced the serum levels of blood urea nitrogen (BUN) and serum creatinine (SCr) along with activity of superoxide dismutase. In glomerular tissues, *L. lucidus* ameliorated the expansion of the mesangial area. Furthermore, *L. lucidus* reduced mRNA levels of TGF-β1 and phosphorylation of SMAD2. Thus, the above study confirmed that *L. lucidus* may be a potential new candidate in inhibiting renal fibrosis by blocking the signaling pathway of TGF-β. The study also points towards a protective effect of *L. lucidus* in preventing renal damage in STZ-induced diabetic rats by (Yao et al., 2013).

#### Puerarin

Puerarin is an isoflavonoid found in many plants and a common adjuvant therapy in China in the alleviation of diabetes, cardiac fibrosis, angina pectoris, and cardiovascular diseases (Zhang et al., 2015; Yuan et al., 2016; Jin et al., 2017). In a study, the effect of puerarin on renal damage was observed in STZ-induced diabetic Wistar rats. The animals were grouped into normal control group, untreated diabetes group, two diabetes groups treated with two doses of puerarin at 140 and 200 mg/kg, respectively and a standard drug-treated group. It was found that the diabetic untreated group compared to the normal control group showed increased levels of total cholesterol, triglyceride, blood glucose, IFN (interferon)-γ, IFN-γ/IL-4, malondialdehyde (MDA), and kidney index while the levels of catalase (CAT), superoxide dismutase (SOD), fasting blood insulin (FPI), body weight, IL-4, glutathione peroxidase (GSH-Px), and nitric oxide (NO) were decreased in the untreated diabetic group. Additionally, the untreated diabetic group displayed increased glomerular extracellular matrix (relative area), Scr, urine protein (UP), and BUN in comparison to the control group. Protein and mRNA expressions of SMAD2, TGF-β1, connective tissue growth factor (CTGF), and fibronectin (FN) were measured using western blot analysis and real-time fluorescence quantitative polymerase chain reaction analysis (RT-FQ-PCR). All these parameters were found to increase in diabetic rats without any treatment as compared to the normal control group. In the puerarin treated group, the elevated parameters were decreased and the decreased parameters were increased in comparison to the diabetes untreated group. Also, there was an improvement in the renal functions in the puerarin treated group as compared to the diabetic untreated group. There was also the downregulation of CTGF, FN, SMAD2, and TGF-β1 mRNA and protein expressions. Thus puerarin exerted its antidiabetic action through inhibitory effects on the TGF-β1/SMAD2 pathway (She et al., 2014).

#### Propolis

Propolis is a natural mixture is found in plants and is produced by bees through the mixing of salivary enzymes and wax with plant material. Mounting evidence has been supported the anti-cancer, anti-bacterial, anti-fungal, anti-viral, anti-inflammatory, immunomodulatory and hepatoprotective properties of propolis (Banskota et al., 2001; Gülçin et al., 2010; Sawicka et al., 2012; Chan et al., 2013). A study was conducted by researchers on the effect of propolis in enhancing the healing of cutaneous wounds in STZ-induced diabetic mice. When untreated diabetic mice were compared with non-diabetic mice, it was shown that a delay in wound closure was found in diabetic mice in comparison to the non-diabetic mice. Additionally, the levels of TGF-β1 were found to be decreased in diabetic mice in comparison to untreated mice. Moreover, the levels of inflammatory cytokines and matrix metalloproteinase 9 (MMP9) were enhanced in the wound tissues in diabetic mice in comparison to the untreated mice. This corresponds with decreased production of collagen and phosphorylation of SMAD2 and SMAD3 in wound tissues of diabetic mice. However, when the propolis treated diabetic group was compared to the untreated diabetic mice, it was found that propolis treatment significantly increased closure of diabetic wounds. Importantly, the levels of TNF-α, IL-6, IL-1β, and MMP9 were found to be at normal levels in propolis treated diabetic mice. The propolis treated group also showed an enhanced formation of collagen by promoting TGF-β1/SMAD2,3 signaling in comparison to the untreated diabetic group (Hozzein et al., 2015).

#### *Momordica charantia* L.

*Momordica charantia* L. (MC) or bitter gourd ointment is a traditional medicine with an ethnobotanical survey in India and the Caribbean commonly used in the management of diabetes (Semenya et al., 2012; Talukdar and Hossain, 2014). For instance, MC was used in a study to evaluate its effect on enhancing the healing of wounds in diabetic Sprague-Dawley rats. Streptozotocin was used to induce diabetes, and the rats were divided into normal control group (non-diabetic), diabetic rats (untreated), diabetic rats treated with MC powder, diabetic rats treated with an ointment containing MC, diabetic rats treated with povidone ointment, and diabetic rats treated with ointment base. The excision wound model was used in the study. All the treatments were given for 10 days. The healing of the wound was determined by total protein content, TGF-β expression, histological observations, and the rate of closure of the wound. Results showed that the closure of the wound was delayed in the diabetic groups as compared to the control group. In comparison to the untreated diabetic group, MC ointment treated group showed a significantly faster rate of closure of wound. Also on day 10, MC ointment treated group showed the best wound closure rate in comparison with other diabetic groups that were given treatment. A high level of total protein content and intense expression of TGF-β1 were shown by MC ointment treated group. The diabetic wound healing potential of MC ointment in the study was suggested to be due to its ability to enhance the expression of TGF-β (Hussan et al., 2014).

#### Curcumin

Curcumin is a polyphenol extracted from *Curcuma longa* L. (Turmeric) as a yellow pigment component which used as a hepatoprotective drug in traditional Chinese medicine (Kumar and Sakhya, 2013). It is known to possess multiple therapeutic actions and has the potential to become an important antidiabetic agent. Protection against diabetic neuropathy through curcumin induced by inhibiting collagen IV, fibronectin and TGF-β1. Curcumin has also shown the potential to reduce diabetic cardiomyopathy. Fibrosis in tissues of heart was found to be decreased by administration of curcumin in a dose of 300 mg/kg/day for 16 weeks in STZ-induced diabetic rats. Also in a clinical study, diabetic patients received 66.3 mg of curcumin per day for 2 months. No changes were made in the medications that they were taking. Results of the trials showed that curcumin did not produce any changes in the lipid or glucose profile, but levels of urinary IL-8 and TGF-β were found to be reduced. TGF-β and IL-8 are involved in the formation of diabetic kidney disease. Thus, curcumin showed potential in lowering complications associated with diabetes (Rivera-Mancía et al., 2018).

#### Hesperidin

Hesperidin (3,5,7-trihydroxy flavanone-7-rhamnoglucoside) is a flavanone glycoside as an active ingredient of citrus fruits with anti-oxidant, neuroprotective, and anti-inflammatory properties (Parhiz et al., 2015) Hajialyani et al., 2019). In a study on the wound healing potential of hesperidin, streptozotocin was used to induce diabetes in Sprague Dawley rats in a dose of 55 mg/kg. The wound was made in the hind paw of rats, and when this wound was stabilized, hesperidin was administered in doses of 25, 50, and 100 mg/kg p.o. for the time duration of 21 days. Several histopathological, molecular, and biochemical parameters were evaluated in the wound tissues of rats. The study showed that hesperidin treatment enhanced vasculogenesis and angiogenesis through upregulation of the Ang1/Tie-2, TGF-β1, SMAD-2/3, and VEGF-c mRNA expression to result in acceleration of healing of the wound in rats (Li et al., 2018).

#### Silymarin

The flavonoid silymarin is a cocktail of flavonolignans obtained from the plant *Silybum marianum* and is used to treat a wide variety of liver problems and diabetes (Rasool et al., 2014; Ferenci, 2016; Belwal et al., 2020; Singh et al., 2020). A study was conducted to study the potential of silymarin in improving diabetic cardiomyopathy by inhibiting TGF-β1/SMAD signaling. In one study, the treatment of diabetic rats with silymarin down-regulated the levels of blood glucose and produced improvements in collagen deposition and cardiac fibrosis in diabetic rats. Cardiac dysfunction in diabetic rats was decreased by silymarin as detected from the results of echocardiography. Silymarin produced a decrease in the levels of TGF-β and p-SMAD2/3 and enhanced the levels of SMAD7 in comparison to the untreated diabetic rats. Thus, this study points towards the potential of silymarin in improving diabetic cardiomyopathy by inhibiting TGF-β1/SMAD signaling. Silymarin can be a potential new candidate for the treatment of diabetic cardiomyopathy (Meng et al., 2019).

## Is There Any Cross-Talk Between TGF-β and Autophagy Signaling Pathways in Diabetes?

TGF-β by using several numbers of signaling pathways except to SMADs can regulate a wide array of cellular processes. As yet, the cross-talk between TGF-β and autophagy has not been studies in diabetes. However, some studies conducted to unravel the involvement of two signaling pathways in progression of diabetic complications such as liver and kidney fibrosis. Combinatory interactions of type I and type II TGF-β receptor serine/threonine kinases induce receptor-activated SMADs and its downstream signaling pathways (Derynck and Zhang, 2003) or directly activate many receptor pathways in a SMAD-independent manner. The PI3K-AKT-mTOR axis is activated directly by TGF-β ligand in a SMAD-independent way, resulting in the phosphorylation of numerous substrates, such as S6 kinase by mTORC1 and AKT by mTORC2, which are important for malignant progression (Ao et al., 2006; Lamouille and Derynck, 2007; Lamouille et al., 2012) and pathological bone metastases. As mTOR activity is tightly associated with many aspects of tumorigenesis, its regulation may be effective in the prevention of tumorigenesis (Zoncu et al., 2011).

TGF-β-miR-96 signaling pathway in an SMAD-dependent manner regulates the activation of mTOR activity, which is under the direct effect of the TGF-β pathway. Indeed, the AKT substrate modulated by mTORC2, AKT1S1 (also known as PRAS40) (Sancak et al., 2007; Vander Haar et al., 2007), is targeted by microRNAs activated in response to TGF-β ligand. Consequently, mTORC1 kinase will protect from the inhibitory effect of AKT1S1 as phosphorylation levels of S6K increase. In addition to AKT1S1, there are several tumor suppressors, such as FOXO1 and FOXO3a (Lin et al., 2010), that are negatively regulated by miR-96. In this line, miR-96 was considered as a metastamir and/or oncomir in the progression of cancer.

Specifically, autophagy targeting of misfolded proteins or damaged organelles improves cell survival (Massagué, 2000; Gozuacik and Kimchi, 2004). However, excessive autophagic activity may lead to type II programmed cell death, which completely differs from apoptosis or type I programmed cell death (Ding et al., 2010). In mouse mesangial cells (MMC), serum deprivation induces autophagy, which eventually results in apoptosis. Yet, treatment with TGF-β results in the induction of the autophagy pathway, suppressing apoptosis activation. In primary MMCs cultured in serum deprivation circumstances, TGF-β signals through the TGF-β-activated kinase 1 (TAK1) and triggers the activation of several downstream cell signaling cascade, including MKK3/6-p38 MAPK and the PI3K-Akt-mTOR-S6K signaling axis (Ninomiya-Tsuji et al., 1999; Wang et al., 2001). The recruitment of the PI3K-Akt pathway (Chen et al., 2007; Gingery et al., 2008; Ding et al., 2010) leads to the induction of autophagy, which inhibits caspase 3 activity. Moreover, under the influence of the PI3K-Akt pathway, the G1/S cell cycle progress through the upregulation of cyclin D1 (Diehl et al., 1998) and downregulation of p27KIP1 (p27) levels through ubiquitination-dependent proteolysis (Murillo et al., 2001). In addition, Blocking TGF-β by autophagy inhibitor in MMC decreases the p27 protein level, suggesting that p27 levels were regulated through autophagy.

Liver fibrosis is a chronic liver disease with augmentation of ECM proteins, especially collagen, in liver tissues (Wu et al., 2017). The process of liver fibrosis is initiated by the activation of hepatic stellate cells (HSCs) (Li et al., 2016). When various factors, such as mechanical stimulation and inflammatory cytokines, especially TGF-β1, activate the quiescent HSCs, HSCs could not preserve the balance between ECM production and degradation (De Minicis et al., 2007). Indeed, TGF-β1 ligand secreted by KCs and HSCs promotes continuous activation of HSCs and interacts with TGF-β receptors (TβRs) of HSCs to phosphorylate SMAD3 and promote the translocation of phospho-SMAD3 (p-SMAD3) to the nucleus, leading to the production of ECM components that facilitate fibrosis pathogenesis (Shi and Massagué, 2003; Su et al., 2014).

The secretion of TGF-β1 could be regulated by the nuclear factor-kappa B (NF-κB) signaling pathway (Hayden and Ghosh, 2008) and activation of NF-κB could be modulated by TGF-β ligand. In other words, binding of IκBα to NF-κB subunits forms the IκBα/p50/p65 complex, which blocks NF-κB translocation into the nucleus (Luedde and Schwabe, 2011). TGF-β1 induced the degradation of IκBα, resulting in the enhancement of NF-κB (13) and also promoted the activation of NF-κB by TGF-β–activated kinase TAK1 and the IκB kinase (Sakurai et al., 1999; Attisano, 2001). In turn, NF-κB induces the transcription of TGF-β1 to promote the activation of HSCs ECM for the development of liver fibrosis (Feng et al., 2015). Furthermore, TGF-β1/SMAD3 pathway can lead to the induction of Beclin-1, which plays a critical role in the nucleation of the autophagy process (Gordy and He, 2012; Li et al., 2016), contributing indigestion of lipid droplets and supplies energy for the promotion of HSCs, thereby developing liver fibrosis (Hernández-Gea et al., 2012). Regarding the pivotal role of the TGF-β1/SMAD3 signaling pathway in activated HSCs, it seems that inhibition of this pathway could be beneficial in treatment of liver fibrosis. In a recent study, salidroside in combination with rat mesenchymal stem cell transplantation showed an efficiency in the treatment of liver fibrosis (Ouyang et al., 2010). In another study, bleomycin-induced lung fibrosis was improved by the administration of salidroside through modulation of the NF-κB and TGF-β1/SMAD2/3 pathways (Tang et al., 2016). In addition, utilizing salidroside was reported to be associated with reduced levels of TGF-β1 in KCs and HSCs *via* suppression of the NF-κB pathway, indicating that reduced autophagy in HSCs was performed by downregulation of the TGF-β1/SMAD3 pathway (Feng et al., 2018).

Fucoidan is another drug which functions similar to salidroside so that by inhibition of TGF-B -β1, reduces phosphorylation of SMAD2/3 and impedes the transferring of SMAD2/3 from the pulp to the nucleus to combine with specific DNA sequences of Beclin-1 gene which is a component of the class III phosphatidylinositol 3-kinase (PtdIns 3-kinase) complex. Therefore, Beclin-1 could not promote the autophagosome biogenesis through interaction with PI3K, which induces the conversion of LC3-I to LC3-II (Gordy and He, 2012; Nikoletopoulou et al., 2013) and consequently hinders the formation of autophagosomes.

In kidney injury and fibrosis induced by a unilateral ureteral obstruction (UUO), deficiency of autophagic protein LC3 and Beclin 1 leads to increased mature TGF-β levels and collagen deposition. In contrast, through autophagic degradation, the mature TGF-β1 protein levels are regulated, and kidney fibrosis induced by UUO will be suppressed (Pang et al., 2016).

Another key mechanism involved in the pathogenesis and progression of kidney fibrosis is the deregulation of epithelial-mesenchymal transition (EMT) (Koesters et al., 2010; Hernández-Gea et al., 2012). It has been shown that autophagy mediates the effect of TGF-β on the induction of EMT (Böttinger and Bitzer, 2002; Masszi et al., 2004; Zheng et al., 2009; Pang et al., 2016). Indeed, TGF-β induces autophagy degrading E-cadherin, resulting in β-catenin release. Following the β-cathenin release, autophagy activates Src in order to phosphorylate β-catenin, which by translocation to nucleus and binding to SMAD2 or SMAD3 as a co-activator increases the gene expression of ILK signaling factor which contributes in EMT. However, it has been demonstrated that both ILK and p-B-catenin/pSMAD2 are able to induce EMT and fibrosis, individually (Kim et al., 2009; Ulsamer et al., 2012; Tian et al., 2013).

It is conceivable that the regulation of autophagy by TGF-β signaling was done in a context-specific manner. Namely, autophagy may be possessed angiogenic or anti-angiogenic effects under various conditions. TGF-β has been connected to autophagy induction through TAK1 and JNK in epithelial and tumor cells, whereas activation of AKT and mTOR mediated by TGF-β has been shown to strongly inhibit autophagy in fibroblasts. In the endothelial cells, TGF-β suppresses transcription of beclin1 mediating PIP3-dependent recruitment of additional ATG proteins through the recruitment of SMAD2. Indeed, SMAD2 functions as an inhibiting factor of Beclin 1 in the autophagy process. In addition, it has been shown that beclin1 gene expression is controlled by several transcription factors such as FoxO3, NFκB, HIF1α, c-Jun, and E2F1 signaling factors.

Extensive studies over the past several years have revealed that autophagy acts as a cytoprotective effector in a response to increased stress so dysregulation of autophagy is found in the development of human disorders (Mizushima et al., 2008; Choi et al., 2013). The culmination of these observations shed light on the importance the interplay of two pathways’ cross-talk and development of several diseases, while further studies is required to uncover the involvement of TGF-β and autophagy cross-talk in pathogenesis of diabetes and rising complication so that paved the way to discover novel therapeutic strategies.

## Concluding Remarks and Perspective

Current strategies for the management of diabetes include the prevention of glucose absorption as well as the inhibition of related metabolic pathways and factors such as gluconeogenesis and α-glucosidase. Moreover, various signaling pathways are involved in the regulation of metabolic disorders, leading to more recent studies to investigate the role of signaling pathways in the normal function of β cells and insulin-responsive cells. Specifically, the main effective signaling pathways, namely, autophagy and TGF-β1/SMAD signaling cascades have been considered as the useful therapeutic strategies in the overcome DM.

Thus, the prominent roles of both autophagy and TGF-β1/SMAD signaling pathways in diabetes and its complications were further supported by altered expression and activity of signals. Furthermore, therapeutic strategies aiming to regulate TGF-β1/SMAD signaling and autophagy present promising remedies in the treatment of diabetes.

## Author Contributions

All authors contributed equally to this article.

## Conflict of Interest

The authors declare that the research was conducted in the absence of any commercial or financial relationships that could be construed as a potential conflict of interest.

## Glossary

**Table:**

3-MA	3-methyladenine
AD	Alzheimer disease
adFNDI	autosomal-dominant familial neurohypophyseal diabetes insipidus
ADSCs	Adipose tissue-derived stem cells
ADT2DM	advanced duration of type 2 diabetes mellitus
ALK	activin receptor-like kinase
AMPK	5′ adenosine monophosphate activated protein kinase
ATF6	activating transcription factor-6
ATG	AuTophaGy related proteins
Atg7	autophagy-related gene 7
Bcl2	B-cell lymphoma 2
BUN	blood urea nitrogen
CAT	catalase
CNS	central nervous system
CTGF	connective tissue growth factor
DDIT3	DNA-damage inducible transcript 3
DM	Diabetes mellitus
DN	diabetic neuropathy
DRP1	Dynamin‐related protein 1
ECM	extracellular matrix
EGFR/MAPK	epidermal growth factor receptor/mitogen activated protein kinase
EIF2AK3	eukaryotic translation initiation factor 2 α kinase 3
EMT	epithelial mesenchymal transition
ER	endoplasmic reticulum
ERN1/Ire1α	endoplasmic reticulum to nucleus signaling 1/inositol requiring enzyme-1α
Ex-4	exendin-4
FADD	Fas-associated death domain protein
FGF21	fibroblast growth factor 21
FPI	fasting blood insulin
FIP200	family interacting protein of 200 Kd
FN	fibronectin
GABA	γ-aminobutyric acid
GABARAPL1	GABA A receptor-associated protein like 1
GLP-1	glucagon-like peptide-1
GLP-1R	glucagon-like peptide-1 receptor
GLUT 4	glucose transporter 4
GS	glycine-serine-rich
GSH-Px	glutathione peroxidase
GSK3	Glycogen synthase kinase 3
GTP	guanosine triphosphatases
hIAPP	human islet amyloid polypeptide
HIF1α	hypoxia inducible factor 1-α
HIHG	high insulin/glucose
HSC	hepatic stellate cell
GDM	gestational diabetes mellitus
iAs	Inorganic arsenic
IR	insulin receptor
pIRE1	IRE1 phosphorylation
JNK	c-Jun N-terminal kinase
LC3	light chain 3
LTBP-1	latent transforming growth factor beta binding protein -1
LAMP-2	lysosome associated membrane protein2
LC3	1A/1B-light chain 3
LCIIIB	light chain IIIB
MHC	major histocompatibility complex
MAPK	mitogen-activated protein kinase
MC	*Momordica charantia*, MDA, malondialdehyde
MFN2	Mitofusin-2
MIN6	mouse INsulinoma 6
MMC	mouse mesangial cells
MMP9	matrix metalloproteinase 9
mRNA	messenger ribonucleic acid
mTOR	mTORC2, rapamycin complex 2
mammalian target of rapamycin	
NAC	N‐acetylcysteine
NAFLD	nonalcoholic fatty liver disease
ND	non-diabetic
NF-kB	nuclear factor kappa-B light-chain-enhancer of activated B cells
NDT2DM	newly diagnosed type 2 diabetes mellitus
NO	nitric oxide
NOD	nonobese diabetic
NPC1L1	Niemann-Pick C1-like
OLETF	Otsuka Long-Evans Tokushima Fatty
PEG-sTNF-RI	pegylated soluble tumor necrosis factor receptor type 1
PPARγ	peroxisome proliferator‐activated receptor γ
PBMCs	peripheral blood mononuclear cells
PKA	protein kinase A
pGDM	prior history of gestational diabetes mellitus
PERK	endoplasmic reticulum kinase
PI3P	phosphatidyl inositol-3 phosphate, Rab, Ras-associated binding
RT-FQ-PCR	real time fluorescence quantitative polymerase chain reaction analysis ROS, reactive oxygen species
rpWAT	retroperitoneal white adipose tissues
scWAT	subcutaneous white adipose tissues
SCr	serum creatinine
SNARE	SolubleN-ethylmaleimide-sensitive factor-attachment protein receptor; reactive oxygen species
SOD	superoxide dismutase
STZ	Streptozotocin
SRX	sulfiredoxin
T2DM	type 2 diabetes mellitus
TAK1	beta-activated kinase 1
TEM	transmission electron microscopy
TGF-β	transforming growth factor β
TNF-α	tumor necrosis factor-α
TGN	trans-Golgi network
TRAF6	Tumor necrosis factor receptor-associated factor 6
TUDCA	tauroursodeoxycholic acid
ULK1	Unc-51-like kinase 1
UP	urine protein
UPR	unfolded protein response
UUO	unilateral ureteral obstruction
VEGF	vascular endothelial growth factor
Vps34	Vacuolar Protein Sorting Protein 34. WIPI, WD-repeat protein interacting with phosphoinositides.
